# Correction to *Lancet Child Adolesc Health* 2019; 3: 245–54

**DOI:** 10.1016/S2352-4642(19)30203-2

**Published:** 2019-08

*Culver LD, Orkin MF, Campeau L, et al. Improving lives by accelerating progress towards the UN Sustainable Development Goals for adolescents living with HIV: a prospective cohort study.* Lancet Child Adolesc Health *2019; **3**: 245–54*—This Article should have been published under a CC BY Open Access license. This correction has been made to the online version as of June 12, 2019.

